# Lifestyle and metabolic factors affect risk for meningioma in women: a prospective population-based study (The Cohort of Norway)

**DOI:** 10.3389/fonc.2024.1428142

**Published:** 2024-08-12

**Authors:** Anamaria Gheorghiu, Cathrine Brunborg, Tom B. Johannesen, Eirik Helseth, John-Anker Zwart, Markus K. H. Wiedmann

**Affiliations:** ^1^ Department of Neurosurgery, Bagdasar-Arseni University Hospital, Bucharest, Romania; ^2^ Faculty of Medicine, University of Oslo, Oslo, Norway; ^3^ Centre for Biostatistics and Epidemiology, Research Support Services, Oslo University Hospital, Oslo, Norway; ^4^ Cancer Registry of Norway, Norwegian Institute of Public Health, Oslo, Norway; ^5^ Department of Neurosurgery, Oslo University Hospital, Oslo, Norway; ^6^ Department of Research and Innovation, Division of Clinical Neuroscience, Oslo University Hospital, Oslo, Norway

**Keywords:** meningioma, metabolic syndrome, parity, diabetes, obesity, brain tumor, risk factor, smoking

## Abstract

**Background:**

Meningioma is the most common primary brain tumor, with a clear preponderance in women. Obesity is considered a risk factor for the development of meningioma. Obesity is also the clinical hallmark of metabolic syndrome, characterized by glucose intolerance, dyslipidemia, and hypertension. Lifestyle and metabolic factors directly impact overweight and obesity and are therefore potential risk factors for meningioma development. The aim of this study is to assess lifestyle and metabolic factors for meningioma risk in women.

**Methods:**

The Cohort of Norway (CONOR) is a nationwide health survey, conducted between 1994 and 2003, including anthropometric measures, blood tests, and health questionnaires. Linkage to the National Cancer Registry enabled the identification of intracranial meningioma during follow-up until December 2018.

**Results:**

A total of 81,652 women were followed for a combined total of 1.5 million years, and 238 intracranial meningiomas were identified. Increasing levels of physical activity (HR 0.81; 95% CI 0.68–0.96; p trend <0.02) and parity (HR 0.83; 95% CI 0.71–0.97; p trend <0.03) were negatively associated with meningioma risk. Diabetes mellitus or glucose intolerance increased the risk for meningioma (HR 2.54; 95% CI 1.60–4.05). Overweight and obesity were not associated with meningioma risk, nor was metabolic syndrome. However, participants without metabolic dysfunction had a reduced meningioma risk, while participants with all five metabolic factors present had a 4-fold risk increase for meningioma (HR 4.28; 95% CI 1.34–13.68).

**Conclusion:**

Lifestyle factors seem to significantly influence meningioma risk. However, disentangling the complex associations and interactions between factors for meningioma risk will be a challenging task for future studies.

## 1 Introduction

In 2016, the International Agency for Research on Cancer (IARC) announced for the first time that avoiding overweight and obesity has a direct influence on the risk for the most common primary CNS tumor, meningioma (1). Our group later questioned the *direct* association between meningioma risk and increased body fatness due to more recent data from the largest prospective cohort study to date (2). However, increased body fatness is accompanied by substantial metabolic and endocrine dysregulation, reflected in changes of sex hormone metabolism, inflammatory pathways, insulin and insulin-like growth factor (IGF) signaling, and adipokines (3). We therefore hypothesize that the assumed association between overweight, obesity, and meningioma risk is merely one aspect of a more complex association involving multiple metabolic factors, such as hyperlipidemia, glucose intolerance, hypertension, and increased body fatness. Furthermore, meningioma shows a strong preponderance for women, making it the tumor with the highest sex predilection among non-sex-specific tumors (4, 5). Although the reason for this remains unclear, hypotheses have included the expression of progesterone and estrogen receptors in meningioma, tumor growth under progesterone agonist therapy, or in women using hormone replacement therapy or oral contraceptives (6, 7). The previous factors may additionally be influenced by different lifestyle and socio-economic factors. To our knowledge, no cohort study to date has integrated all these aspects into one study. The aim of this study was therefore to assess the risk of meningioma in a large prospective cohort study of adult women, considering not only body fatness but also different metabolic and lifestyle factors.

## 2 Materials and methods

### 2.1 Ethical statement

This study was approved by the Regional Committee for Ethics in Medical Research (REK No 2011/428).

### 2.2 Study population

The Cohort of Norway (CONOR) is a health survey that describes the population of Norway regarding the distribution of exposures and health status, considering anthropometric measures, blood tests, health questionnaires, lifestyle, and socio-economic factors. Contributing regional health surveys all agreed on about 50 core CONOR questions, and the first CONOR survey was conducted in Tromsø in 1994 (8). Enrolment of participants in CONOR occurred between 1994 and 2003. Detailed information about the study and the exact wording of the questions are available on the CONOR website (https://www.fhi.no/en/studies/conor/about-conor—data-from-several-regional-health-studies/). In all CONOR surveys, letters of invitation were sent 2 weeks before the appointment, including a questionnaire and information about the study. At the time of screening, participants underwent a physical examination, and blood samples were collected. Supplementary questionnaires were handed out to the study participants to be returned by mail in a pre-addressed and stamped envelope (8).

Body weight and height were measured according to a standard protocol. Heart rate, systolic, and diastolic blood pressures were measured by an automatic device after 2 min of seated resting, and three recordings were made at 1-min intervals.

In total, 309,742 persons were invited to the CONOR survey between 1994 and 2003. The overall participation rate was 56%.

### 2.3 Linkage of databases

Norwegian residents have a unique 11-digit ID number universally used for personal identification. This number enabled the linkage of the CONOR participants to the Norwegian Cancer Registry for identification of any tumor diagnosis during follow up. Furthermore, linkage to the Norwegian Tax Administration allowed identification of all study participants’ status (i.e., resident, emigrated, deceased) at the last follow-up (15.12.2018). The Norwegian Cancer Registry is a national cancer registry with mandatory reporting by clinicians and pathology departments since 1952.

### 2.4 Outcome characteristics

Based on the International Classification of Diseases for Oncology, third edition (ICD-O-3), meningioma was defined as 9530-9539. To define intracranial location, morphology codes were combined with topography codes 193.0–193.2 and 195.3–195.5 based on the International Classification of Diseases, Seventh Revision (ICD-7). Meningiomas included were diagnosed by histopathology or imaging only.

### 2.5 Categorization of independent variables

Height and weight of study participants were measured by trained personnel at study baseline. BMI was calculated as weight divided by height squared (kg/m^2^) and categorized as <20 kg/m^2^, 20 kg/m^2^–24.9 kg/m^2^, 25 kg/m^2^–29.9 kg/m^2^, and ≥30 kg/m^2^ as well as per 5 kg/m^2^ increase in BMI. Overweight was defined as BMI 25 kg/m^2^–29.9 kg/m^2^, obesity as BMI ≥30 kg/m^2^, underweight as BMI <20 kg/m^2^ and BMI 20 kg/m^2^–24.9 kg/m^2^ was used as the reference category. Civil status was categorized as married or partner, single, widow, and separated. Education was categorized as 0 years–10 years, 11 years–15 years, and over 15 years. Physical activity level was based on the survey questions on the extent of *regular* physical activity per week as defined by the total time of activity per week in hours (none, <1, 1–2 or ≥3) and the intensity of activity (light activity; not sweating or out of breath, and hard activity; sweating or out of breath). Activity levels were defined as: None, Low activity level (1 h–2 h of light activity, no hard activity), moderate activity (<1 h of hard activity ± 1 h–2 h light activity or ≥3 h of light activity alone), high activity level (minimum 1 h–2 h of hard activity or up to 1 h per week of hard activity plus minimum 2 h of light activity).

Smoking was categorized into daily cigarette smoking at present, previously, and never. Alcohol consumption was divided into once per month or less, two to three times per month, once per week or several times per week on a regular basis. Reproductive variables considered were age at menarche (≤12 years, 13 years, 14 years, and >14 years), parity (0, 1, 2, or ≥3 children), or menopausal status.

Hypertension was defined as either mean systolic blood pressure (BP) ≥140 or diastolic ≥90 or use of anti-hypertensive drugs. The continuous variables systolic or diastolic BP and blood lipids (cholesterol, triglycerides, HDL, LDL) were divided into quartiles. LDL was calculated based on the Friedewald formula. Metabolic syndrome definition was based on the Adult Treatment Panel III (ATP III) criteria (9), i.e., the presence of a minimum three of the following five traits: Abdominal obesity, (defined as BMI ≥30 kg/m^2^); serum triglycerides ≥1.7 mmol/L or drug treatment for elevated triglycerides; serum HDL cholesterol <1.3 mmol/L or drug treatment for low HDL-C; BP ≥130/85 mmHg or drug treatment for elevated blood pressure; fasting plasma glucose (FPG) ≥5.6 mmol/L or drug treatment for elevated blood glucose.

### 2.6 Definition of follow-up time

Follow-up time was calculated as person-years from the time of the baseline study examination (measurement of BMI, blood pressure, and blood sampling) until the date of meningioma diagnosis, any other cancer diagnosis, date of emigration, date of death from any cause, or the end of follow-up on 15 December 2018, whichever occurred first.

### 2.7 Statistical analysis

Cox proportional hazard regression, using attained age at study baseline as the time axis, was performed to calculate hazard ratios (HR) with 95% confidence intervals (CI). Risk factors for meningioma included in multivariable analyses were selected based on prior knowledge from the literature, pre-defined ATP III criteria for metabolic factors, and if identified by univariable analysis, while carefully considering multi-collinearity. Multi-collinearity between risk factors was assessed using Spearman`s correlation coefficient model ≥0.7 as the cut-off. To account for multiple hypothesis testing in our Cox proportional hazards regression models, we applied the Benjamini–Hochberg procedure to reduce the false discovery rate.

Sensitivity analysis was performed to minimize the likelihood of reverse causality regarding BMI by excluding participants with 5 or less years of follow-up. The proportional hazard assumptions were tested by plotting the logarithm of the integrated hazards (log–log survival plots) and by Schoenfeld tests. A two-sided probability with a significance level of 0.05 was used throughout. Statistical analyses were performed with STATA/SE statistics software Version 17 (StataCorp, 4905 Lakeway Dr, TX 77845, USA).

## 3 Results

### 3.1 Characteristics of the study population

The final study cohort consisted of 81,652 women between 20 years and 80 years of age, after excluding participants with any tumor diagnosis before baseline screening (6,464), age >80 (3,143), pregnancy at the time of screening (1,302), or missing measurements for BMI (503), blood lipids (356), and blood pressure (147).

At study inclusion, 26% of the study participants were under 40 years of age. Median follow-up time was 19.8 years (IQR 16.7–22.3) and total follow-up was 1,498,626 years. During follow-up, 249 meningiomas were identified, of which 238 were located intracranially. A total of 97 (39%) were diagnosed by imaging only, while the remaining were diagnosed by histopathology. Details of the baseline characteristics are provided in **Table 1**.

**Table 1:** Characteristics of the population at risk and intracranial meningioma cases in CONOR.

	Population at risk	Meningioma
No of participants	81,652	238
Mean age at study baseline	48 (SD 14.4)	51 (SD 13.2)
BMI (kg/m^2^)		
<20	4,649	12
20–24.9	37,181	90
25–29.9	27,054	93
≥30	12,768	43
Married (%)	59.8	61.8
Hypertension (%)	30.5	41.2
Diabetes (%)	3.6	8.4
Metabolic syndrome (%)	11.3	13.5
LDL >4.1 mmol/L or statin use (%)	30.7	38.6
Parity (no child) (%)	13.7	13.5
Hard physical activity ≥2 times per week	33.5	25.6
Current smoker (%)	31.5	30.6
Alcohol consumption ≥2 times per week (%)	7.9	3.4
Education >15 years (%)	14.8	14.7

### 3.2 Socio-economic and life-style factors

Neither civil status, length of education, nor smoking was associated with the risk of meningioma (**Table 2**). Increasing levels of physical activity (HR 0.85; 95% CI 0.74–0.99) in trend analysis and alcohol consumption (HR 0.84; 95% CI 0.73–0.97) were associated with a reduced risk of meningioma (**Table 2**).

**Table 2:** HRs (95% CIs) for lifestyle and reproductive factors and risk for intracranial meningioma among women in CONOR (univariable analyses).

	No. at risk	Cases	HR (95% CI)
Civil status			
Single	16,621	40	Ref
Married	48,863	147	0.73 (0.50–1.06)
Widow	6,933	22	0.77 (0.43–1.37)
Separated	9,125	29	0.79 (0.48–1.29)
Missing	110	0	–
Education (in years)			
≦10	43,824	136	Ref
10 to 14	23,266	56	0.99 (0.72–1.36)
≧ 15	12,083	35	1.16 (0.79–1.70)
Missing	2,479	11	1.58 (0.85–2.95)
*p trend*			*1.06 (0.88–1.28) p = 0.523*
Cigarette smoking			
None	49,191	146	Ref
Current	25,739	73	1.00 (0.75–1.33)
Previous	5,926	17	1.13 (0.68–1.86)
Missing	796	2	0.86 (0.21–3.49)
Physical activity level			
Inactive	4,483	15	Ref
Low	16,322	60	1.00 (0.57–1.76)
Moderate	17,434	55	0.88 (0.50–1.57)
High	27,384	61	0.68 (0.38–1.20)
Missing	16,029	47	0.77 (0.43–1.37)
*p trend*			*0.85 (0.74–0.99) p = 0.035*
Regular alcohol consumption			
≦1 times per month	35,480	119	Ref
2–3 times per month	17,871	44	0.76 (0.54–1.08)
Once per week	12,273	40	0.95 (0.66–1.37)
≧2 times per week	6,410	8	0.36 (0.18–0.74)
Missing	9,618	27	0.74 (0.49–1.14)
*P trend*			*0.84 (0.73–0.97) p = 0.019*
Parity			
None	11,204	32	Ref
1	10,272	28	0.77 (0.46–1.28)
2	25,538	71	0.66 (0.43–1.01)
≧3	27,329	80	0.62 (0.41–0.95)
Missing	7,309	27	0.84 (0.50–1.41)
*p trend*			*0.86 (0.76–0.99) p = 0.034*
Age at menarche in quartiles			
1.	20,923	67	Ref
2.	20,172	47	0.71 (0.49–1.03)
3.	17,963	61	0.98 (0.69–1.39)
4.	14,339	34	0.68 (0.45–1.03)
Missing	8,255	29	0.96 (0.62–1.50)
Menopausal status			
Pre-menopausal	49,817	124	Ref
Post-menopausal	31,835	114	1.04 (0.71–1.53)

Cox regression models with age as the time axis.

HRs, hazard ratios; Cis, confidence intervals; Ref, reference.

### 3.3 Reproductive and hormonal factors

Age at menarche or menopausal status was not associated with the risk of meningioma. However, women with two or more children had a reduced risk of meningioma, and there was a significant trend for risk reduction per child born (HR 0.86; 95% CI 0.76–0.99) (**Table 2**).

### 3.4 Anthropometric measures, blood pressure, and blood lipid levels

BMI, body height, systolic and diastolic blood pressure, blood total cholesterol, triglycerides, and HDL levels were not significantly associated with the risk of meningioma (**Table 3**). Although LDL levels per quartile were not significantly associated with meningioma risk, there was a dose-dependent increased risk (HR 1.14; 95% CI 1.01–1.30 per category).

**Table 3:** HRs (95% CIs) for anthropometric data, blood pressure and blood lipids, diabetes mellitus, hypertension, and risk for intracranial meningioma among women in CONOR (univariable analyses).

	No. at risk	Cases	HR (95% CI)
Height in quartiles			
1.	22,226	73	Ref
2.	18,770	59	0.94 (0.67–1.33)
3.	19,754	53	0.83 (0.58–1.20)
4.	20,902	53	0.84 (0.58–1.22)
*P trend*			*0.94 (0.83–1.05) p = 0.277*
BMI category (kg/m^2^)			
<20	4,649	12	1.22 (0.67–2.23)
20–24.9	37,181	90	Ref
25–29.9	27,054	93	1.31 (0.97–1.75)
≥30	12,768	43	1.30 (0.90–1.87)
*p trend*			*1.12 (0.96–1.31) p = 0.159*
Systolic BP (quartiles)			
1.	20,940	42	Ref
2.	21,111	59	1.28 (0.86–1.91)
3.	20,804	70	1.40 (0.95–2.07)
4.	18,797	67	1.42 (0.93–2.17)
*p trend*			*1.12 (0.98–1.27) p = 0.098*
Diastolic BP (quartiles)			
1.	21,567	53	Ref
2.	20,823	55	0.98 (0.67–1.43)
3.	19,951	58	0.98 (0.67–1.43)
4.	19,311	72	1.16 (0.80–1.68)
*P trend*			*1.05 (0.93–1.18) p = 0.425*
Cholesterol (quartiles)			
1.	21,622	50	Ref
2.	20,394	41	0.74 (0.49–1.12)
3.	19,939	71	1.21 (0.83–1.75
4.	19,697	76	1.22 (0.83–1.81)
*p trend*			*1.12 (0.99–1.27) p = 0.082*
Triglycerides (quartiles)			
1.	21,454	53	Ref
2.	20,825	57	1.03 (0.71–1.50)
3.	19,950	52	0.95 (0.65–1.40)
4.	19,423	76	1.39 (0.97–1.99)
*p trend*			*1.11 (0.98–1.24) p = 0.097*
HDL (quartiles)			
1.	20,925	72	Ref
2.	23,829	62	0.74 (0.53–1.04)
3.	16,985	47	0.78 (0.54–1.12)
4.	19,913	57	0.77 (0.54–1.09)
*p trend*			*0.93 (0.83–1.04) p = 0.182*
LDL (quartiles)			
1.	20,413	41	Ref
2.	20,599	49	1.03 (0.68–1.60)
3.	20,118	69	1.37 (0.92–2.03)
4.	19,482	74	1.42 (0.94–2.13)
*p trend*			*1.14 (1.01--1.30) p = 0.04*
Diabetes or glucose intolerance			
No	77,686	216	Ref
Yes	2,969	20	2.54 (1.60–4.05)
Missing	997	2	0.83 (0.21–3.32)
Hypertension			
No	56,744	140	Ref
Yes	24,908	98	1.42 (1.06–1.91)

Cox regression models with age as the time axis.

HRs, hazard ratios; Cis, confidence intervals; Ref, reference; BMI, body mass index; BP, blood pressure; HDL, high density lipoprotein; LDL, low density lipoprotein.

### 3.5 Metabolic factors and metabolic syndrome

While body mass index, either in categories for overweight and obesity or per 5 kg/m^2^ increase, was not associated with the risk for meningioma (**Table 3**), the metabolic factors diabetes (HR 2.54; 95% CI 1.60–4.05) and hypertension (HR 1.42; 95% CI 1.06–1.91) increased the risk of meningioma (**Table 3**).

**Figure 1:**
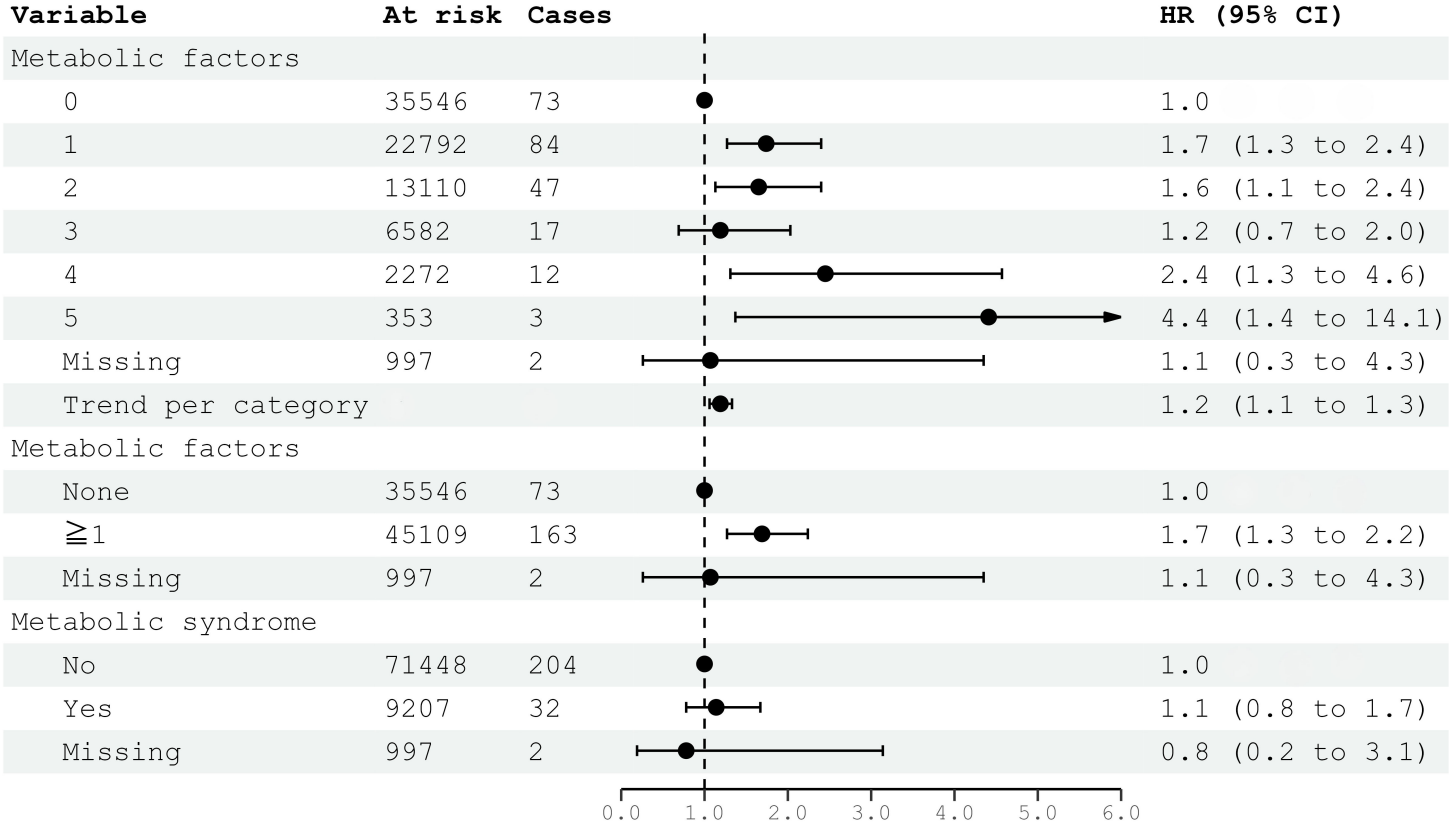
HRs (95% CIs) for metabolic factors and metabolic syndrome and risk for intracranial meningioma among women in CONOR. Multivariable Cox regression model including all five metabolic factors (obesity, HT, increased serum triglycerides, decreased HDL, glucose intolerance or diabetes mellitus) and education. Correlation matrix of coefficients of cox model did not confirm multi-collinearity between variables. HR, hazard ratio; CI, confidence intervals.

Metabolic factors, as defined per ATP III criteria, were associated with the risk of meningioma, especially if four (HR 2.45; 95% CI 1.31–4.57) or five (HR 4.41; 95% CI 1.37–14.14) factors were present (**Figure 1**). However, the risk of meningioma was also increased with only one factor present (HR 1.74; 95% CI 1.27–2.40) (**Figure 1**). The presence of *metabolic syndrome*, as defined by ATP III criteria, requires the clustering of at least three out of five factors. Metabolic syndrome was not significantly associated with the risk of meningioma (HR 1.14; 95% CI 0.78–1.67).

### 3.6 Multivariable analyses

**Figure 2:**
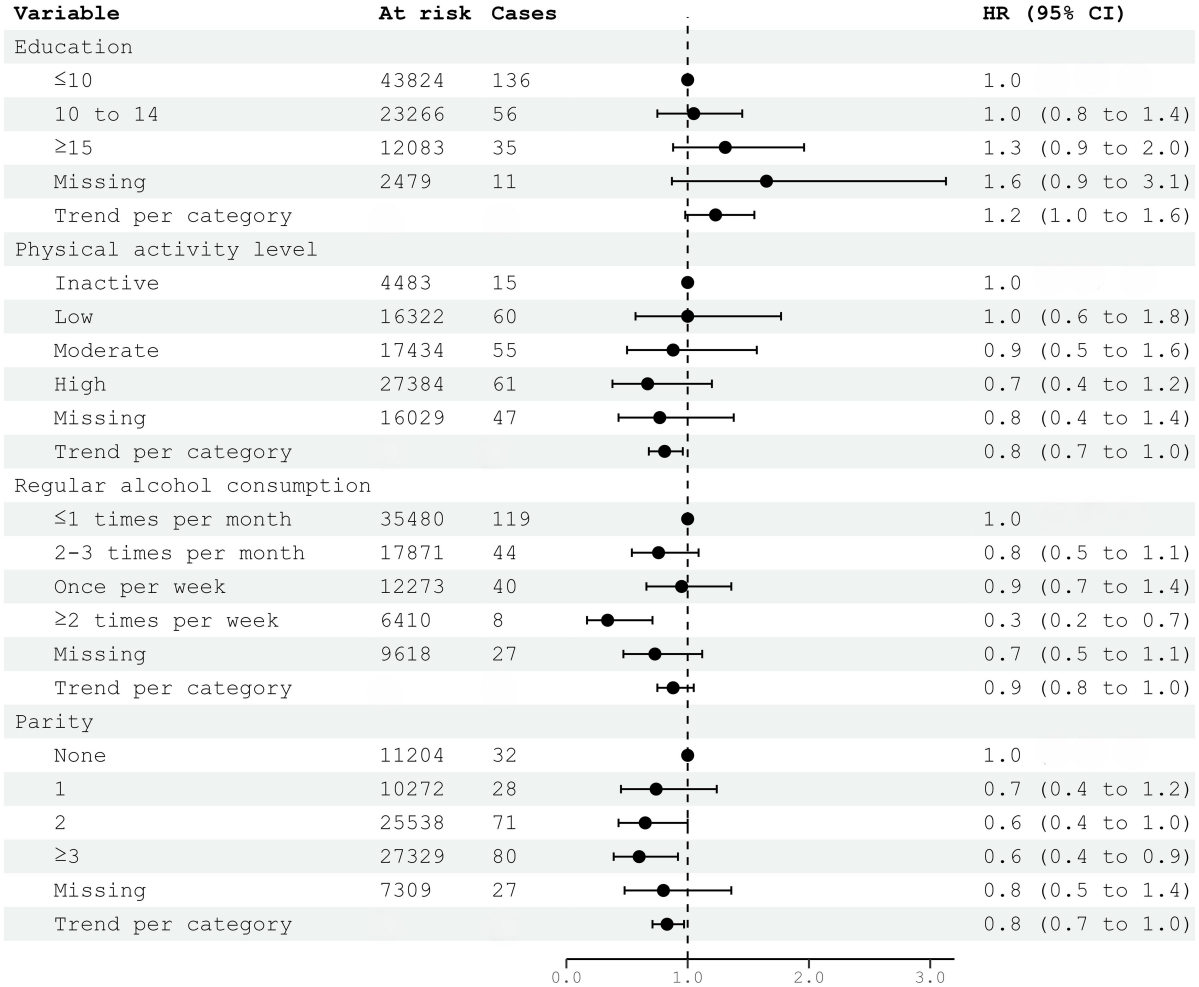
HRs (95% CIs) in multivariable models including education, physical activity, alcohol consumption, parity, and risk for intracranial meningioma among women in CONOR. Cox regression models with age as the time axis. HR hazard ratio; CI confidence intervals. Correlation matrix of coefficients of cox model did not confirm multi-collinearity between variables.

In multivariable models, education was not associated with meningioma risk. Although individual categories of physical activity were not significantly associated, there was a negative trend between increasing physical activity and meningioma risk (HR 0.81; 95% CI 0.68–0.96) (**Figure 2**). The association of parity with reduced meningioma risk also remained significant for two or more children born and as trend (HR 0.83; 95% CI 0.71–0.97) (**Figure 2**). Only the highest level of alcohol consumption (HR 0.34; 95% CI 0.17–0.71), but not the trend per category, was associated with reduced meningioma risk in multivariable analyses (**Figure 2**). The analysis of metabolic syndrome and factors was itself a multivariable model and included education as proxy for socio-economic differences. There was an increase in meningioma risk per category (HR 1.19; 95% CI 1.06–1.33) (**Figure 2**).

### 3.7 Sensitivity analyses

Reverse causality may occur if latent and undetected meningioma is present, potentially influencing independent variables. We therefore excluded the first 5 years of follow-up in sensitivity analyses to reduce this risk of bias. However, in trend analysis, an increasing level of activity remained significantly associated with decreasing meningioma risk (HR 0.83; 95% CI 0.69–1.00) (**Table 4**) The highest category of alcohol consumption also remained significant (HR 0.41; 95% CI 0.20–0.85), while the trend for increasing alcohol consumption was not (**Table 4**). Parity was associated with decreased meningioma risk, both for women with three or more children (HR 0.60; 95% CI 0.38–0.96) and as a trend per category (HR 0.81; 95% CI 0.68–0.96). Diabetes mellitus remained significantly associated with meningioma risk (HR 2.02; 95% CI 1.17–3.50), but not hypertension (**Table 5**). The association between metabolic factors and meningioma risk was weakened, and no significant trend was found. Having one or more metabolic factors was associated with meningioma risk (HR 1.61; 95% CI 1.19–2.18), but not metabolic syndrome (HR 0.87; 95% CI 0.55–1.36) (**Table 5**).

**Table 4:** HRs (95% CIs) in multivariable models including education, physical activity, alcohol consumption, parity, and risk for intracranial meningioma among women in CONOR in *sensitivity analyses* (excluding the first 5 years of follow-up).

	No. at risk	Cases	HR (95% CI)
Education (in years)			
≦10	41,708	117	Ref
10 to 14	22,590	50	1.08 (0.76–1.53)
≧15	11,745	29	1.24 (0.80–1.92)
Missing	2,246	9	1.56 (0.77–3.16)
*p trend*			*1.19 (0.93–1.53) p = 0.159*
Physical activity level			
Inactive	4,114	9	Ref
Low	15,638	55	1.53 (0.75–3.10)
Moderate	16,752	49	1.31 (0.64–2.79)
High	26,639	53	0.98 (0.48–2.02)
Missing	15,146	39	1.05 (0.51–2.18)
*p trend*			*0.83 (0.69–1.00) p = 0.046*
Regular alcohol consumption			
≦1 times per month	34,009	99	Ref
2–3 times per month	17,391	37	0.76 (0.52–1.12)
Once per week	11,848	35	0.98 (0.66–1.45)
≧2 times per week	61,445	8	0.41 (0.20–0.85)
Missing	8,896	26	0.87 (0.55–1.35)
*P trend*			*0.91 (0.76–1.10) p = 0.336*
Parity			
None	10,770	26	Ref
1	9,904	26	0.85 (0.49–1.46)
2	24,669	63	0.70 (0.44–1.11)
≧3	26,034	66	0.60 (0.38–0.96)
Missing	6,912	24	0.87 (0.55–1.35)
*p trend*			*0.81 (0.68–0.96) p = 0.014*

Multivariable Cox regression models with age as the time axis, excluding the first 5 years of follow-up.

HRs, hazard ratios; Cis, confidence intervals; Ref, reference.

**Table 5:** HRs (95% CIs) for diabetes mellitus, hypertension, metabolic factors and metabolic syndrome, and risk for intracranial meningioma among women in CONOR in *sensitivity analyses* (excluding the first 5 years of follow-up).

	No. at risk	Cases	HR (95% CI)
Diabetes or glucose intolerance			
No	74,714	119	Ref
Yes	2,640	14	2.02 (1.17–3.50)
Missing	935	1	0.47 (0.07–3.37)
Hypertension			
No	55,211	129	Ref
Yes	23,078	76	1.13 (0.82–1.56)
Metabolic factors			
0	34,562	65	Ref
1	21,814	76	1.75 (1.25–2.45)
2	12,437	41	1.59 (1.06–2.37)
3	6,179	14	1.07 (0.60–1.93)
4	2,070	8	1.79 (0.85–3.79)
5	292	0	–
Missing	935	1	0.60 (0.08–4.30)
*p trend*			*1.10 (0.97–1.25) p = 0.141*
Metabolic factors			
None	34,562	65	Ref
≧1	42,792	139	1.61 (1.19–2.18)
Missing	935	1	0.60 (0.08–4.33)
Metabolic syndrome			
No	68,813	182	Ref
Yes	8,541	22	0.87 (0.55–1.36)
Missing	935	1	0.44 (0.06–3.13)

Cox regression models with age as the time axis excluding the first 5 years of follow-up.

HRs, hazard ratios; Cis, confidence intervals; Ref, reference.

## 4 Discussion

In this prospective cohort study of women, risk for meningioma was inversely associated with physical activity, parity, and alcohol consumption. Metabolic syndrome, as defined by ATP III criteria (i.e., ≧3 metabolic factors), was not associated with an increased risk for meningioma. Diabetes mellitus or glucose intolerance was robustly associated with increased meningioma risk.

### 4.1 Metabolic factors and meningioma

Metabolic syndrome, characterized by adiposity, dyslipidemia, diabetes or glucose intolerance and hypertension, has previously been associated with increased risk for meningioma (10, 11). Muskens et al. did not find an association between meningioma risk and increased BMI or diabetes in women, but they did not find an association with hypertension (HR 1.21; 95% CI 1.02–1.44). Bernardo et al. could not assess metabolic syndrome in their cohort study but investigated the biomarkers blood glucose level and cholesterol (12). In their study, higher fasting serum glucose levels were inversely associated with meningioma risk in women but not in men, and no association between cholesterol levels and meningioma risk was found (12). In a matched case–control analysis of women and men, Seliger et al. found BMI ≥30 (OR 1.33; 95% CI 1.17–1.52) and arterial hypertension (OR 1.34; 95% CI 1.20–1.49) as factors of metabolic syndrome associated with meningioma risk, but not dyslipidemia or glucose intolerance (10). Seliger et al. investigated medications used to treat metabolic syndrome factors, but did not present a combined variable of glucose intolerance and diabetes. However, a combined metabolic syndrome variable was positively associated with meningioma risk in women (OR 1.44, 95% CI 1.17–1.78) (10). The largest population-based pooled cohort study reporting on metabolic factors and brain tumor risk, found a significant increased risk for meningioma in patients with metabolic syndrome (HR 1.31; 95% CI 1.11–1.54) (11). Furthermore, increased systolic and diastolic blood pressure were positively associated with meningioma risk, but not the effect estimates for BMI, triglycerides, HDL, or glucose levels (11).

Arterial hypertension was also positively associated with the incidence of meningioma in women in the 60 to 69-year age group (OR 2.23; 95% CI 1.03–4.84) in a case–control study (13). In our study, hypertension was significantly associated with increased meningioma risk in univariable analysis, but the effect was attenuated in multivariable models. In our dichotomized variable for hypertension, we also included cases with hypertension under medical treatment. This may bias the study results during long-term follow-up, as adequately treated hypertension may attenuate the effect of high blood pressure on meningioma risk. We therefore cannot rule out that hypertension is a potential risk factor for meningioma. There was also a trend for increased meningioma risk with increasing levels of LDL in our cohort, but the association was not very robust. Again, long-term treatment with statins, which has become common, may attenuate a more robust association, and data need to be interpreted carefully. The effect of diabetes mellitus and glucose intolerance, on the other hand, was robust and the effect size remarkable. It may be speculated that the effect size is even underestimated, as the category of participants with diabetes also includes those who receive treatment.

We did not find a convincing association between BMI and meningioma risk, in conformity with several other studies (2, 14, 15), but not with all (10, 16, 17). This is not likely to be due to just a lack of power, as we found similar results in a very large cohort study with sufficient power (2). However, the proportion of obese and morbidly obese populations may be higher in other countries and cultures, and therefore a potential effect may be underestimated in our cohort.

Interestingly, cases without any metabolic factor at baseline had a reduced risk for meningioma compared to those with one or more factors (HR 1.72; 95% CI 1.30–2.28), and there was a dose–response relationship (HR 1.22; 95% CI 1.09–1.36; per category). Cases with all five metabolic factors present had an over 4-fold risk increase for meningioma (HR 4.28; 95% CI 1.34–13.68). Unfortunately, it is difficult to differentiate the possible influences of metabolic factors and their potential interactions on meningioma risk, as they are naturally entangled.

### 4.2 Lifestyle and reproductive factors

Smoking was previously promoted as a protective factor for meningioma risk in women, due to its anti-estrogenic effect by increasing estradiol metabolism (18). This was not confirmed by our study, which is in congruent with a previous meta-analysis (19).

Regular alcohol consumption more than once per week seemed protective against meningioma risk in our study. However, we did not find a gradient for the increasing number of times alcohol was consumed and the risk for meningioma. Unfortunately, we did not have robust data on the units of alcohol consumed to investigate a reliable dose–response relationship. Drinking habits in Norway have differed from those in other European countries, as drinking alcohol regularly several times per week has not been considered a common cultural habit. We therefore hypothesize detection bias in the subgroups with higher frequency of alcohol consumption, as higher thresholds for doctor visits or the relation of symptoms to alcohol consumption may lead to the underreporting of meningioma. Interestingly, alcohol use (never vs ever) also seemed protective in a case–control study by Claus et al. (OR 0.77; 95% CI 0.64–0.93), but without further elaboration by the authors (20).

Both parity and physical activity seemed protective for meningioma in our study. Muskens et al. did not find a reduced risk for meningioma in parous women compared to nulliparous women. However, there seemed to be a protective effect for parous women in higher age groups at the time of the first live birth (15). In the EPIC cohort study by Michaud et al. no association between parity and meningioma risk was found (21). Additionally, physical activity was not associated with meningioma risk in the EPIC cohort study. In the “Million Women Study” by Benson et al., one of the largest prospective cohort studies, physical activity had a protective effect against meningioma, while smoking, alcohol consumption, socio-economic level, and parity were not associated with meningioma risk (22). In the Nordic-UK brain tumor study, a case–control study by Wigertz et al., reproductive factors were generally not associated with meningioma risk (23). However, when differentiating between the number of pregnancies and the number of life-births given, ≥3 live births were associated with increased meningioma risk in women <50 years of age. The authors argued that this reflects an effect of hormones on tumor growth rather than tumor initiation. As meningiomas are slow-growing, the raised levels of estrogens and progesterone during pregnancy may stimulate the growth of an already existing but asymptomatic tumor. Since no effect of parity was found among older women, this is consistent with the assumption that parity has a promotive effect on already existing meningioma (23). Other reproductive factors, such as age at menarche or menopause, have generally not been associated with meningioma risk, in conformity with our study (20, 21, 23). The predilection of meningioma for the female sex and the expression of sex steroid hormone receptors in the majority of tumors have long promoted expectations in epidemiological research (24). So far, evidence from epidemiological data has generally been weak, although a causative role for estrogens and progestins had been hypothesized. Increased BMI has been promoted as a risk factor for meningioma by the IARC (1), but our group previously questioned the validity of this direct association (2). This doubt has been further supported by another recent study (15). Undoubtedly, adiposity comes with other metabolic risk factors and may therefore be a proxy for other disease mechanisms. At least, our study indicates that diabetes or glucose intolerance, and possibly increasing levels of LDL and hypertension, are associated with increased meningioma risk and that women without any of the metabolic risk factors have a decreased risk for meningioma. DM and glucose intolerance have been the strongest risk factors for meningioma in our study.

### 4.3 Explanatory mechanisms

Metabolic dysfunction is a key risk factor for obesity-related cancer (25). In particular, glucose intolerance represents a risk factor for cancer, including hepatocellular, hepatobiliary, pancreatic, breast, ovarian, endometrial, and gastrointestinal cancers (26). Common risk factors, such as age, obesity, physical inactivity, and smoking, may amplify the increased cancer risk in patients with DM. Although the linkage between diabetes and cancer is not completely understood, the biological mechanisms include hyperglycemia, hyperinsulinemia, increased bioactivity of insulin-like growth factor 1, oxidative stress, dysregulations of sex hormones, and chronic inflammation (26). However, cancer screening rates are significantly lower in people with DM, thus introducing potential detection bias. Also, evidence from previous studies has suggested that some medications used to treat DM have been associated with either *increased* or *reduced* risk of cancer (26). In particular, discoveries about the possible reduced incidence of cancer development in patients treated with metformin, a much used anti-diabetic drug, have forced both endocrinologists and oncologists to reconsider the mechanistic links between diabetes mellitus and cancer (27–30).

The association between metabolic factors, lifestyle, and the development of meningioma may be explained through several biological mechanisms. Meningiomas frequently express estrogen and progesterone receptors, with the latter being present in up to 90% of meningiomas (6, 7, 31, 32). Estrogen and progesterone can promote meningioma growth by binding to their respective receptors and activating transcriptional programs leading to increased cell proliferation and reduced apoptosis (24, 31, 33). Furthermore, metabolic factors such as obesity and DM may increase meningioma risk through chronic systemic inflammation and insulin resistance, which can increase circulating levels of insulin-like growth factors (IGFs) (34–36). Metabolic dysregulation further leads to chronic inflammation, increased oxidative stress and impaired immune function (37, 38). IGFs, particularly IGF-1, bind to their receptors on meningioma cells, activating intracellular signaling cascades such as the PI3K/Akt and Ras/Raf/MEK/ERK pathways, which promote cellular proliferation and survival (39, 40). Other growth factors, such as vascular endothelial growth factor (VEGF) and platelet-derived growth factor (PDGF), are also implicated in meningioma biology, contributing to angiogenesis and further supporting tumor growth (41–43). Lifestyle factors may further influence these hormonal and metabolic pathways, suggesting that the interplay between systemic hormonal environment, metabolic status, and growth factor activity can create a milieu conducive to meningioma development.

### 4.4 Study limitations

This study has several limitations. Not all meningiomas were diagnosed by histopathological examination, but approximately 39% were diagnosed by imaging only. As not all meningiomas need surgical treatment but can be confidently diagnosed by imaging and followed conservatively, we think they should be included in a prospective cohort study on risk factors for the tumor. However, some uncertainty remains as small incidentally found meningiomas may not always be reported to the Cancer Registry, introducing the possibility of ascertainment bias.

Our study assessed risk for meningioma in women only. A significant limitation in understanding sex differences in meningioma lies in the lack of histological confirmation and grading for a large proportion of cases. The changes in the World Health Organization classification of meningiomas in 2016 and 2021 have indeed impacted the diagnosis and categorization of more malignant meningiomas (44, 45). Criteria for grading atypical (WHO Grade 2) and anaplastic (WHO Grade 3) meningiomas were refined to incorporate more precise histological features such as increased mitotic activity and brain invasion (44, 45). Tumors that might have been considered borderline between WHO grades under older criteria may now be more definitively classified as atypical or anaplastic based on these refined parameters. Specific mutations, such as those in the NF2 gene, are more frequently observed in higher-grade meningiomas and are slightly more common in men, suggesting a genetic basis for the observed differences in tumor aggressiveness (46, 47). Progesterone, androgen, and estrogen receptors are commonly expressed in meningiomas, particularly in women (6, 7). The hormonal environment in females may influence tumor growth and behavior differently than in males. Unfortunately, our study data did not allow us to investigate these aspects of meningioma diagnosis.

Study participants diagnosed with diabetes, hypertension, or dyslipidemia are exposed to an increased risk of cerebrovascular disease and may therefore be more prone to cerebral imaging. This may lead to an increased detection rate of meningioma, creating detection bias. Although we found significant associations between different variables and risk for meningioma, it is important to stress that causality cannot be stated. BMI and waist circumference increase slightly with age, and in the case of a small effect size of BMI on meningioma risk, this may remain undetected in studies with long follow-up times. External validity: 99% of our study population were of Caucasian ethnicity, and study results may therefore not be representative of other populations as CNS tumor incidences and risk profiles may vary for different ethnicities (15, 48). Furthermore, we could not exclude hereditary tumor syndromes known to be associated with meningioma risk in our analyses, yet those are usually rare and thus of minor statistical impact. We could not differentiate DM type 1 from DM type 2 in our study. However, DM type 2 accounts for about 95% of DM cases in general, thus representing the vast majority (49). LDL levels in our study were calculated by the Friedewald formula and not directly measured. In prospective, population-based cohort studies, selection or non-response biases are reduced compared to case–control studies. This increases reliability and significance. However, as meningioma incidence is low, cohort studies may result in small numbers, hampering subgroup analyses and increasing the risk of missing important associations.

This is further complicated by the issue of multiple testing, inherent in conducting multiple Cox proportional hazards regression analyses. Although the application of the Benjamini–Hochberg procedure helps to reduce the false discovery rate, it does not completely eliminate the risk of Type I errors. Additionally, any correction method for multiple testing will reduce the power to detect true associations, particularly when the number of tests is large. Therefore, the trade-off between controlling false positives and maintaining statistical power must be carefully considered (50). In our study, correction by the Benjamini–Hochberg method confirmed the effects of diabetes and metabolic factors, while parity, alcohol consumption, and physical activity were just above the critical Benjamini–Hochberg value. These limitations highlight the importance of cautious interpretation of our findings and suggest that further validation in independent cohorts is warranted.

### 4.5 Study strengths

Strengths of this study include the large size of a population-based cohort, encompassing women of a wide range of ages and the long follow-up time. Further, this is the first prospective cohort study with comprehensive data on lifestyle, reproductive, and metabolic factors, thus providing extensive information on potential risk factors and confounders. Height, weight, and blood pressure were measured in a standardized fashion by trained personnel, and blood tests for blood lipid and glucose analyses, including fasting time, were conducted. Follow-up was virtually complete due to linkage to the National Cancer Registry of Norway and the Norwegian Tax Administration. The National Cancer Registry provides high quality incidence data for tumors, including completeness and validity (51).

## 5 Conclusion

This comprehensive prospective cohort study provides additional evidence and insight into the risk factors for meningioma development. Lifestyle factors appear to significantly influence meningioma risk. However, disentangling the complex associations and interactions between risk factors for meningioma will be a challenging task for future studies.

## Data Availability

The data that support the findings of this study are available from the Cancer Registry of Norway and the Norwegian Institute of Public Health (https://www.fhi.no/en). Restrictions apply to the availability of these data, which were used under license for this study. Requests to access these datasets should be directed to folkehelseinstituttet@fhi.no.
